# Exploring Functional Differences between the Right and Left Ventricles to Better Understand Right Ventricular Dysfunction

**DOI:** 10.1155/2021/9993060

**Published:** 2021-08-28

**Authors:** Judith Bernal-Ramirez, Magda C. Díaz-Vesga, Matias Talamilla, Andrea Méndez, Clara Quiroga, Javier A. Garza-Cervantes, Anay Lázaro-Alfaro, Carlos Jerjes-Sanchez, Mauricio Henríquez, Gerardo García-Rivas, Zully Pedrozo

**Affiliations:** ^1^Tecnológico de Monterrey, Escuela de Medicina y Ciencias de la Salud, Ave. Morones Prieto 3000, Monterrey, NL 64710, Mexico; ^2^Programa de Fisiología y Biofísica, Instituto de Ciencias Biomédicas, Facultad de Medicina, Universidad de Chile, Santiago de Chile, Chile; ^3^Advanced Center for Chronic Diseases, Facultad de Ciencias Químicas y Farmacéuticas & Facultad Medicina, Universidad de Chile, Santiago de Chile, Chile; ^4^Centro de Estudios en Ejercicio, Metabolismo y Cáncer (CEMC), Facultad de Medicina, Universidad de Chile, Santiago de Chile, Chile; ^5^Red para el Estudio de Enfermedades Cardiopulmonares de Alta Letalidad (REECPAL), Universidad de Chile, Santiago de Chile, Chile; ^6^Grupo de Investigación en Ciencias Básicas y Clínicas de la Salud, Pontificia Universidad Javeriana de Cali, Colombia; ^7^Escuela de Kinesiología, Facultad de Salud y Ciencias Sociales, Campus Providencia, Sede Santiago, Universidad de las Américas, Chile; ^8^Centro de Investigación e Innovación Biopsicosocial en Enfermedades Crónicas, Facultad de Salud y Ciencias Sociales, Universidad de las Américas, Chile; ^9^División de Enfermedades Cardiovasculares, Facultad de Medicina, Pontificia Universidad Católica de Chile, Santiago, Chile; ^10^Tecnológico de Monterrey, Centro de Investigación Biomédica, Hospital Zambrano Hellion, TecSalud, San Pedro Garza Garcia, NL 66278, Mexico

## Abstract

The right and left ventricles have traditionally been studied as individual entities. Furthermore, modifications found in diseased left ventricles are assumed to influence on right ventricle alterations, but the connection is poorly understood. In this review, we describe the differences between ventricles under physiological and pathological conditions. Understanding the mechanisms that differentiate both ventricles would facilitate a more effective use of therapeutics and broaden our knowledge of right ventricle (RV) dysfunction. RV failure is the strongest predictor of mortality in pulmonary arterial hypertension, but at present, there are no definitive therapies directly targeting RV failure. We further explore the current state of drugs and molecules that improve RV failure in experimental therapeutics and clinical trials to treat pulmonary arterial hypertension and provide evidence of their potential benefits in heart failure.

## 1. Introduction

Pulmonary arterial hypertension (PAH) is an incurable life-limiting disease characterized by increased pulmonary hypertension secondary to pulmonary vasculature remodeling [1]. The increased pressure overloads the right ventricle (RV), inducing adaptative RV remodeling. In the initial stages, RV hypertrophy decreases wall tension, but maladaptive remodeling induces RV dysfunction and right heart failure syndrome in the end stages [2]. Specific treatment includes therapies targeting endothelin, nitric oxide, and prostacyclin pathways in pulmonary arteries to decrease pulmonary pressure and prevent RV stress [3]. The available therapeutic approaches improve quality of life and reduce the incidence of clinical worsening [4]. Although RV dysfunction and the patient's response to PAH-specific treatment determine survival [5, 6], there are no therapeutic aims to improve RV dysfunction [7]. Left ventricular (LV) dysfunction mechanisms have been widely studied, and multiple therapies to improve LV failure survival are available [8]; however, treatment for RV dysfunction is less robust [9]. Notably, beta-blockers and drugs that target the renin-angiotensin-aldosterone system (RAAS), which are standard therapies for LV failure, are potentially contraindicated in RV dysfunction [8]. Thus, understanding the differences between the RV and LV and describing RV dysfunction's underlying mechanism may be essential to outline an RV-directed therapy and improve PAH patient outcomes. This review focuses on the underlying mechanisms that differentiate left and right ventricles in both physiological conditions and disease development.

## 2. Structural and Functional Differences between the Right and Left Ventricles

The heart is a muscular pump whose primary function is to supply blood to the body, allowing oxygen and nutrients to reach each cell while removing carbon dioxide and metabolic waste. The ventricles propel blood from the heart to either high-pressure systemic circulation by the thick-walled conic-shaped LV or pulmonary circulation by the thin-walled, crescent-shaped RV, which is capable of maintaining low pressure levels even under changes in volume [10, 11]. Both ventricles adapt their mechanisms at the cellular and tissue levels to meet the whole organism's needs and their development into adulthood to accomplish the heart's function. This section summarizes the differences in development and adaptations of each ventricle to maintain its proper function.

### 2.1. Structural Differences between Ventricles

Embryonic development of the human cardiovascular system occurs between the third and eighth weeks of gestation [12]. Specifically, heart development begins on the 16th day of gestation; however, it is not a uniform process. Ventricles show differences in development, cellular origin, and molecular and genetic markers. These differences begin with the movement of cardiac progenitor cells that originate in gastrulation, from the mesoderm to the anterior of the primitive vein [13], where two structures are differentiated: the first cardiac field (FHF) and the second cardiac field (SHF) [14]. The FHF will give origin to the crescent-shaped cardiac tube and the LV, which begins development before the RV. The SHF will give origin to the outflow tract and the RV. It is essential to note that these processes develop successively and under genetic control, including the Paired-Like Homeodomain 2 (*PITX2*) gene, which determines left and right asymmetry [13], and the Heart and Neural Crest Derivatives Expressed (*HAND1* and *HAND2*) genes, which influence the development of the left and right ventricles, respectively, [14]. Contrary to what happens in adulthood, where cardiac output is the same for both ventricles, during embryological development, the RV produces 60% of total cardiac output [11]. Likewise, during embryological development, the thickness and strength generated by the LV and RV are the same [12].

An organ's structure serves its function; thus, differences in the pressure of pulmonary and systemic circuits determine several structural differences between ventricles. Noting the anatomical muscle arrangement in both ventricles helps to understand how blood is pumped through different parts of the circulatory system. Most of the muscle fibers in the RV free wall are transverse fibers with a small portion of subendocardial longitudinal fibers [15]. However, the LV is composed of endocardial and epicardial fibers, which form a helical structure, and circumferential fibers located at the midwall [16]. Therefore, RV needs fewer muscle fibers and is much thinner than the LV, and it has about one-third of LV's thickness [10]. This fiber arrangement contributes differently to ventricle contraction. LV contraction involves the septum, presenting a radial constriction and longitudinal shortening, contributing 67% and 33% to the LV ejection fraction (LVEF), respectively [16]. Simultaneously, longitudinal fibers in the RV free wall account for 20–30% of the RV ejection fraction (RVEF). In comparison, approximately 80% of RV systolic function is attributed to the septum's helical fibers, which twist and shorten the longitudinal axis in the RV [15]. Along with differences in fiber arrangement and muscle contraction, the RV has a higher extracellular matrix content than the LV [17].

### 2.2. Physiological Difference between Ventricles

Anatomical differences between the ventricles are also reflected in their perfusion system. The lower pulmonary arterial pressure and pulmonary vascular resistance are 20% and 10% of systemic arterial pressure and systemic vascular resistance, respectively [18], leading to lower oxygen consumption by the RV [19]. While the LV has a higher oxygen demand, its perfusion predominantly occurs during diastole due to the fact that increased intramural pressure during systole impedes the flow supply [19]. The low pressures handled by the RV allow the perfusion of blood flow throughout the entire cardiac cycle, allowing it to maintain an appropriate myocardial oxygen level [19]. Moreover, the collateral vessels of the RV are denser than those of the LV [10]. The lower oxygen consumption and blood flow in the RV result in an oxygen extraction reserve, making the RV less vulnerable to myocardial ischemia [19]. However, the RV is highly susceptible to acute increases in afterload, unlike the LV [20, 21]. Increases in pulmonary arterial pressure increase intramural pressure, impeding blood supply during systole, which increases blood flow demands during diastole, like LV perfusion [19]. After the blood flow fails to meet an acute or chronic increased oxygen demand caused by an increased afterload, it results in RV ischemia and RV failure [19, 22].

### 2.3. Differences in Cell Shortening and Relaxation between Cardiac Cells

The cardiac muscle's functional unit is the cardiomyocyte, whose primary function is to accomplish the cell contraction-relaxation cycle, leading to synchronized organ contraction and relaxation [23]. This synchronization is made possible by cardiac excitation-contraction coupling (ECC), which is the physiological process of converting an electrical stimulus to a mechanical response [24]. ECC refers to everything from the activation of the calcium ion (Ca^2+^) transient by initial membrane depolarization, through the action potential (AP), to myofilament contraction in response to increased intracellular Ca^2+^. The initial AP promotes the entry of extracellular Ca^2+^ through voltage-dependent Ca^2+^ channels at the plasma membrane or sarcolemma, which promotes the release of Ca^2+^ from the sarcoplasmic reticulum (SR) in a process known as calcium-induced calcium release (CICR), causing a significant transient increase in intracellular Ca^2+^ [24], which interacts with the proteins in myofilaments to produce cellular contraction. Cell relaxation occurs by removing cytosolic Ca^2+^ in a highly energy-dependent process [25]. This section will focus on describing the differences between left and right cardiomyocytes during ECC, especially the differential characteristics of AP, components of Ca^2+^ handling in myocytes, and energetic and mitochondria-dependent process in excitation energetic coupling.

**Figure 1:**
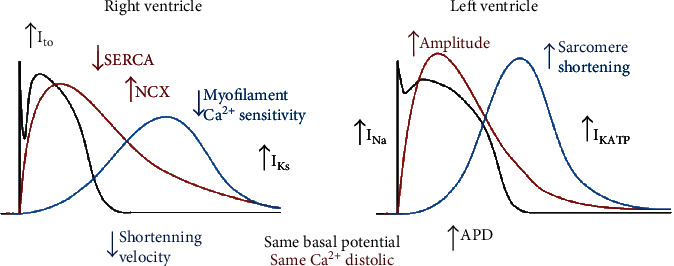
Physiological differences in excitation-contraction coupling between ventricles. Black lines, letters and arrows represent the action potential; red lines, letters and arrows represent Ca^2+^ transient; blue line, letters and arrows represent cellular shortening. *I*_to_: transient outward current; *I*_Ks_: slowly activating component; *I*_Na_: sodium inward current; *I*_KATP_: ATP-activated K^+^ current; ADP: action potential duration; SERCA: sarcoendoplasmic reticulum Ca^2+^ ATPase; NCX: sodium-calcium exchanger. The figure was created with BioRender.com.

By definition, AP involves a reversible change in membrane potential due to the sequential activation and inhibition of several ionic channels, which allow ions to flow in favor of their electrochemical gradient through the cell membrane [26]. Sodium ion (Na^+^) and Ca^2+^ inward currents and different potassium ion (K^+^) outward currents are described in this section. Differences in AP form and duration (APD) are explained by changes in the expression and function of these ions' channels (Table 1).  Figure 1 highlights the main differences between the right and left AP shape and currents. Membrane depolarization by AP starts with a sodium inward current (*I*_Na_) through voltage-sensitive Na^+^ channels. Higher *I*_Na_ densities and larger Na^+^ currents have been found in the LV than in the RV. In the LV, Na^+^ channels also have more negative steady-state inactivation, *V*_1/2_, and slower recovery from inactivation than in the RV, without changes in the activation threshold [26]. The lower *I*_Na_ density causes a slower conduction time in the RV, resulting in a lower upstroke velocity [26]. Despite the lower density, higher [27] or unchanged [26] Na^+^ channel expression has been reported.

The movement of different ions through the cell membrane shapes the AP, organizing it in well-defined membrane depolarization and repolarization phases. The main difference between LV's and RV's AP is during phase 1, which corresponds to the synchronized opening of K^+^ channels after the initial Na^+^ inward current [28, 29]. The RV has a deeper notch than the LV due to an increase in outward K^+^ current density [28, 30, 31]. This increase is due to the larger amplitude of the transient outward current (*I*_to_) in the RV than in the LV [28, 30–32]. In some studies, no changes were observed in protein expression [31, 33] or in the inactivation constant [30, 32]. APD differences between the LV and RV have been described in several species, with some studies finding more prolonged APD in the LV than the RV [29, 30, 32, 34–36], even in human hearts [37]. However, a lack of changes in APD was reported in Langendorff-perfused guinea pig hearts [38], and 2-9% RV longer APD has been observed in dogs [26]. The K^+^ repolarization currents can explain the shorter APD present in the RV. The RV's steeper repolarization phase's significant contribution is partially due to a higher density in the RV of the slowly activating component (*I*_Ks_) of the delayed rectifier *K*1 current [32]. In contrast, a rapidly activating component (*I*_Kr_), the inward rectifier current (*I*_K1_), and the sustained current (*I*_SS_) do not show changes in expression, density, or inactivation [29–33]. The ATP-activated K^+^ current (*I*_KATP_) has been identified as a determinant factor of APD in ischemia, and its expression is higher in the LV than in the RV [38].

Changes in AP duration and shape may be considered since the cardiac AP's immediate consequence is the generation of an intracellular Ca^2+^ transient and differences observed between the APs of the LV and RV may influence intracellular Ca^2+^ dynamics. The initial membrane depolarization triggers the activation of L-type Ca^2+^ channels (LTCC), allowing an inward current of Ca^2+^, which, in turn, promotes the release of Ca^2+^ from the SR through the ryanodine receptors (RyR) by CICR, originating the Ca^2+^ transient [24].  Figure 1 shows the main differences between the RV and LV in the Ca^2+^ transient.

The link between the initial membrane depolarization and the Ca^2+^ transient is the LTCC. There is a clear difference between the AP in both ventricles; however, the initial phase of the Ca^2+^ transient is not affected by these changes. Indeed, while some reports show an increase in LTCC protein expression in the RV [27], others report unchanged gene expression between ventricles [29]. Moreover, the Ca^2+^ currents (*I*_Ca_) do not show differences between ventricles [29].

Regarding RyR, there are no differences in Ca^2+^ concentration for half-maximal activation, the Hill coefficient, caffeine-sensitive ryanodine binding, or current density [39]. However, there are discrepancies in RyR expression in the RV, since some studies show unchanged protein expression, while others refer to lower expression [40]. More studies will be required to clarify these discrepancies.

At rest, there is no difference in diastolic Ca^2+^ between the right and left ventricles [29, 41]. However, although it seems that RyR expression and function are unchanged, it has been reported an increase in Ca^2+^ transient amplitude during systole in the LV [29, 42], indicating a major Ca^2+^ release by the SR due to primary Ca^2+^ content [42]. A higher contraction force [36] and greater sarcomere shortening have been found in the LV than in the RV [29, 36, 41, 43], which coincides with the increase in the transient amplitude since the more significant the Ca^2+^ release, the greater the contraction force. However, two previous studies found no changes in sarcomere shortening in rats [41, 42]. Furthermore, at the molecular level, actin interacts differently with myosin cross-bridges in the LV, allowing greater mobility of actin monomers and, hence, greater contractility [44, 45], without changes in troponin I and T, myosin-binding protein C (MyBP-C), or the myosin regulatory light chain phosphorylation, between LV and RV [46]. On the other hand, the maximal shortening velocity is also slower in RV myocytes [29], which is related to decreased Ca^2+^ sensitivity in RV myofilaments [46–48]. However, greater myosin ATPase activity [49, 50] and a faster cellular contraction in the RV have also been reported due to a larger proportion of heavy *α*-chain-containing myosin isozyme in the RV compared to the LV, which has a larger proportion of the slower *β*-chain [49]. All the expression changes between ventricles are summarized in Table 1.

For relaxation to occur during diastole, intracellular Ca^2+^ must decline, and the sarco/endoplasmic reticulum Ca^2+^-ATPase (SERCA) pump is the primary removal mechanism [24]. As illustrated in Figure 1, a more prolonged Ca^2+^ transient has been reported in RV myocytes than in LV myocytes [41, 42], accompanied by decreased SERCA activity [41, 42] and expression, as well as affinity to Ca^2+^ in the RV [41]. Phospholamban (PLB) is a critical SERCA inhibitor, but PLB phosphorylation relieves SERCA of its inhibition [51]. A previous study found that LV and RV present similar SERCA/PLB ratios but the RV's SERCA-PLB complex is more stable than in LV [41]. The decreased SERCA activity in RV myocytes may allow more active participation of other Ca^2+^ removal mechanisms, leading to lower Ca^2+^ availability in the SR. This phenomenon might explain the decreased transient amplitudes and SR content [42] observed in RV myocytes when compared to LV myocytes. However, there are some discrepancies since faster relaxation has been reported in the RV [36] than in the LV, as well as no differences in SERCA and PBL activity [29] and expression [27, 42] between LV and RV.

Another important Ca^2+^ removal mechanism in cardiomyocytes is the Na^+^/Ca^2+^ exchanger (NCX). Higher NCX protein expression has been found in RV than in LV [27] (Table 1), which might also explain the decreased SR Ca^2+^ availability, resulting in a decreased Ca^2+^ transient amplitude without changes in SERCA activity. However, regardless of its expression, NCX is more active in LV than in RV [36], promoting Ca^2+^ overload in the SR during the rest-potentiation phenomenon, which is more prominent in the LV than in the RV [36]; thus, there are differences in the balance between Ca^2+^ entry and SR loading in the right and left ventricles. Notably, the mitochondrial Ca^2+^ uniporter and mitochondrial NCX contribute to Ca^2+^ handling in cardiac cells [25], but the function and expression of these systems remain unknown in RV cardiomyocytes.

Otherwise, cell relaxation is a high energy-dependent process. Ca^2+^ removal against its concentration gradient by SERCA and the detachment of myosin heads from actin require an adequate ATP supply [24]. Mitochondria are the organelle responsible for energy production in ATP form. There is no change in respiratory components, oxidative metabolism, fatty acid oxidation, or mitochondria respiration between the right and left ventricles [52, 53]. However, the LV has a higher rate of oxidation and mitochondrial membrane potential. This finding has been understood as higher mitochondrial content, supported by a higher citrate synthase activity [53], a higher mitochondria-myofibril ratio [54], and higher nitrosylated protein content in LV than in RV [53]. The mechanism that induces differential levels of mitochondrial biogenesis between the LV and RV is entirely unknown and could be a fertile research area in the future.

## 3. Distinctions between Right and Left Ventricle Dysfunction

In the vascular system, the RV has not received much research attention since 1943, when cauterization of the RV free wall in canine hearts did not change venous pressure [55]. Furthermore, the LV is more severely affected than the RV in heart disease. However, the medical field's perception of the RV is changing from it being considered unimportant to it being an essential component of normal hemodynamics [56]. More recently, significant differences have been recognized in right and left heart failure progression [11]. Although changes in the left ventricles of failing hearts have been thoroughly described, the assumption that the same mechanism is involved in LV and RV failure has been challenged in recent decades. Physiological and structural differences between the two ventricles may explain the differences in the pathologies each ventricle faces, giving importance to underlying mechanisms that make them more susceptible or resistant to diverse insults.

### 3.1. Differences between Right and Left Ventricular Infarction

The compromised coronary artery predominantly determines the size and location of the infarction. Acute right ventricular infarction (RVMI) can occur when there is occlusion of the right coronary artery (RCA), proximally to the takeoff of RV branches [57]. The RVMI is an infrequent event, occurring in one-third to one-half of patients presenting with inferior myocardial infarction; very rarely, it can occur in isolation [58].

The term RV infarction may be somewhat misleading since acute RV ischemic dysfunction frequently has a faster recovery than LV infarction. Indeed, there is a deep contrast between the effects of ischemia and reperfusion in RV and in LV, in which prolonged ischemia often leads to myocardial infarction. Levin and Goldstein proposed diverse reasons to explain LV's lower vulnerability to infarction. First, oxygen demand is undoubtedly lower in the RV than in the LV, because of its much smaller muscle mass and lower afterload. Second, in the absence of severe RV hypertrophy or pressure overload, the coronary artery flow in the RV is given in both diastole and systole. Third, chronic RV failure attributable to RV myocardial infarction is infrequent. Fourth, there is greater availability of blood perfusion in the RV through the collateral flow from the left to right coronary arteries [59]. However, Heresi et al. used a sensitive assay to measure cardiac troponin I (cTnI), a myocardial infarction biomarker, and found a significant positive association between cTnI and a more severe PAH and worse clinical outcomes in patients with PAH [60], suggesting that the susceptibility of the RV to ischemic events is not completely understood.

### 3.2. Differential Mechanisms of Right versus Left Pathological Remodeling

Cardiac hypertrophy is defined as an increase in cardiac mass manifested by increasing size, as well as morphological and functional alterations attributed to a physiological or pathological stimulus. Physical exercise is an example of a physiological stimulus, while a pathological stimulus is found in hypertension, diabetes, myocardial ischemia, and other conditions [61]. Cardiac hypertrophy is considered an adaptive response to increased activity or functional overload, and it is classified as eccentric or concentric. An increase in preload due to high blood volumes reaching the heart, usually observed in aortic regurgitation or endurance exercise, leads to eccentric hypertrophy. This represents a serial addition of sarcomeres, which increases of the ventricular chamber volume and the wall thickness. A higher afterload due to pressure overload in the ventricle leads to concentric hypertrophy. This represents a parallel addition of sarcomeres, which increases myocardial thickness and reduces the diameter of the ventricular chamber [61].

Concentric hypertrophy is generally accompanied by remodeling to adapt to pressure overload and maintain a stable cardiac output. Next, remodeling progresses from an adaptive to a maladaptive phenotype, with altered contractility that leads to cardiac failure [62]. Differences in LV and RV responses to pressure overload have been described [63, 64]. The compensatory remodeling is restricted in RV versus LV. Inhibition of nitric oxide with L-NAME generates LV and RV hypertrophy, but the RV responds with dilation, dysfunction, and an increase in reactive oxygen species (ROS), which causes the inhibition of hypoxia-inducible factor 1-*α* (HIF1*α*) and the suppression of angiogenesis, inducing chronic ischemia in the RV [64, 65]. Moreover, a reduction in superoxide dismutase in the RV versus its increase in the LV has been observed [64]. Additionally, pressure overload in the RV in pulmonary artery banding (PAB) models leads to higher mortality and oxidative stress than pressure overload in the LV by aortic constriction. PAB models also produce more elevated hypoxia in the RV after surgery, with less capillary density and ischemia [66].

Furthermore, mechanical stress on the ventricular wall due to pressure overload stimulates fibroblasts to differentiate into myofibroblasts that produce type II and III collagen in the LV and RV, contributing to cardiac failure [67, 68]. However, differences in the distribution of extracellular matrix (ECM) protein and metalloproteinases in the LV and RV may be explained by a further ECM degradation pattern between ventricles [69], which could explain why effective antifibrotic therapies in LV failure are not effective in RV failure [70]. On the other hand, in chronic thromboembolic pulmonary hypertension, to adjust the RV afterload and wall stress, RV pathological remodeling and wall hypertrophy occur [71], and ECM biomarkers, such as matrix metalloproteinases 2 and 9, decrease, while tissue inhibitor of metalloproteinases-1 (TIMP-1) increases significantly [72]. Notably, treatment with a massive pulmonary embolus is applied to relieve the RV afterload (e.g., pulmonary artery endarterectomy, systemic thrombolytics, or percutaneous intervention), resulting in significant regression of pathological remodeling and RV hypertrophy [71].

Studies have shown shared molecular pathways to hypertrophy and fibrosis between the LV and RV, such as TGF-*β*, Rho-ROCK, and MAPKs. However, differences in signaling have been observed in MAPKs [61, 65]. Phosphorylated p38 (p-p38) MAPK increases in RV fibroblasts and mediates fibrosis induced by TGF-*β* and ventricular dysfunction; however, hypertrophy and changes in proinflammatory genes are not mediated by p-p38 MAPK [73]. In the LV, the role of p38 MAPK, specifically p38*α*, has been also described as a mediator of fibrosis and hypertrophy, but conversely, interleukin-6 is involved as a probable pathway to induce hypertrophy [74]. Furthermore, while the apelin receptor (APJ) participates in hypertrophy induced by pressure overload and apelin prevents hypertrophy in the LV [75–77], the role of APJ in RV has not been elucidated [78].

Difference between RV and LV responses also depends on the stimulus. ROCK signaling mediates hypertrophy induced by metabolic alterations in the LV and by hypoxia in the RV [61], but also, it induces hypertrophy in the LV and RV in pressure overload models, inducing p-ERK1/2 and GATA4 [66, 79]. Studies have demonstrated angiotensin II's role through AT1R in LV hypertrophy due to pressure overload [80–82], whereas an increase in mRNA levels of angiotensin in the monocrotaline (MCT) model has been reported [83]; however, its role in RV has not been fully demonstrated. Angiotensin II is also involved in the induction of autophagy [81]. The role of autophagy in cardiac hypertrophy is controversial; however, basal autophagy would be essential for the preservation of cellular homeostasis, whereas excessive autophagy or its inhibition could aggravate hypertrophy. In different models, such as LV hypertrophy induced by pressure overload or metabolic dysfunction [84, 85] and RV hypertrophy induced by monocrotaline-induced pulmonary arterial hypertension (MCT-PAH) or hypoxia [86, 87], hypertrophy would be mediated by the induction of autophagy, while its inhibition could prevent hypertrophy [85].

On the other hand, activation of proteasome has been observed in LVs exposed to pressure overload, which produces hypertrophy [88], similar to findings obtained in RVs [89]. However, another study performed using the pressure overload model in RVs observed a reduction in proteasome activity [90]; this study was conducted 8-10 days after surgery contrarily to the previous study performed three weeks after surgery [89].

Epigenetic mechanisms have also been identified in cardiac hypertrophy. Class I histone deacetylase inhibitors (HDACs) induce hypertrophy in the LV and RV [91, 92], whereas class IIa HDACs prevent hypertrophy [91, 93]. Unlike findings in the LV [94], inhibitors of HDACs aggravate RV hypertrophy induced by pressure overload [93, 95]. Therefore, further studies are needed to better understand hypertrophy mechanisms in the LV and, mainly, in the RV.

The inflammatory response also plays an essential role in heart failure progression by the activation of proinflammatory cytokines [96]. The increase in inflammatory mediators that can interfere with cardiac contractility and remodeling in PAH correlates to RV dysfunction [97]. While the effects of anti-inflammatory therapies in LV failure are unclear [98, 99], they might be useful in preventing RV failure since perivascular inflammation triggers RV inflammation in a vicious cycle that leads to RV failure [97].

### 3.3. An Overview of the Similarities and Differences between Right and Left Ventricular Failure

The increase in LV afterload by an overload of pressure or volume is considered a determining cause of left heart failure (LHF). In contrast, pulmonary hypertension, pulmonary stenosis, chronic obstructive pulmonary disease, and tricuspid valve pathology produce similar consequences on the right side, inducing right heart failure (RHF) [100–102]. RHF could be acute or chronic. Acute RHF is caused by a suddenly increased RV afterload due to hypoxia or a pulmonary embolus [102, 103] or decreased RV contractility in RV ischemia, myocarditis, or postcardiotomy shock [104]. On the other hand, chronic RHF results from the gradual increases in RV afterload produced by pulmonary hypertension [101, 102], which promotes cardiac remodeling with increased RV mass, fibrosis, and hypertrophy of cardiomyocytes, analogous to the remodeling observed in LHF [105].

As a further example of the interconnection between both ventricles, the prevalence of RV dysfunction increases with LHF progression [106], and RV function and RV–pulmonary artery coupling fail progressively across HF stages [107]. In a community-based cohort study, subclinical RV dysfunction was present in nearly 20% of elderly people and was associated with common HF risk factors. Among people without HF, lower RVEF was associated with HF and death independent of LVEF or N-terminal pro-brain natriuretic peptide (pro-BNP) [107], suggesting that RV dysfunction plays a crucial and underestimated role in HF progression. RV dysfunction was observed in 48% [108] and 33% [109] of heart failure with reduced ejection fraction (HFrEF) and with preserved ejection fraction (HFpEF) patients, respectively, and HFpEF patients displayed greater right-sided chamber enlargement, higher RV diastolic pressure, and more severe contractile dysfunction compared to controls [109].

Furthermore, in patients with LHF, the development of pulmonary hypertension and RV dysfunction is common, and they play an essential role in disease progression, morbidity, and mortality. The diagnosis of pulmonary hypertension aggravates the prognosis in HFpEF and HFrEF patients, and pulmonary hypertension is observed in approximately 75% of patients with HFpEF. Thereby, this prevalence is higher than in patients with HFrEF [100, 110]. In a large community-based prospective cohort of 1,049 subjects with HF, pulmonary hypertension was described as an independent and strong predictor of mortality [110]. Pulmonary hypertension was also defined as a decisive factor in posttransplant mortality because the significantly elevated levels of pulmonary vascular resistance in the postoperative period to which the donor's heart is exposed could trigger RV dysfunction [111].

The requirements of oxygen, glucose absorption, and the glycolytic rate increase in both the LV and RV, reducing fatty acid metabolism [112]. An increased hemodynamic load causes the activation of a pattern of early response or the immediate-early genes c-fos and c-jun, followed by the induction of a “fetal gene program” for the sarcomeric proteins and natriuretic peptides: atrial natriuretic peptide (ANP) and BNP, whose expression is observed also in both ventricles [66, 113].

Mitochondrial dysfunction is an important and crucial mechanism in the development of heart failure [114]. Hypertrophy triggers the Warburg effect in the RV, shifting metabolism from aerobic to anaerobic, showing a decrease in glucose oxidation and increased uncoupled glycolysis and glucose uptake [115], as well as decreasing mitochondrial membrane potential and compromising ATP production [116]. Therefore, protecting mitochondrial function and metabolism has shown positive results in preserving RV function. On the other hand, glutamine antagonist [117] and sodium-glucose cotransporter 2 (SGLT2) inhibitors [118] positively affect cardiac performance, RV hypertrophy, and survival. Regarding fatty acid oxidation (FAO), the information is controversial since RV function improvement has been observed following FAO inhibition [119] and stimulation [120]. Improvement in mitochondrial fragility and membrane potential by activating SIRT3 through stilbene resveratrol administration improves RV function and decreases fibrosis and hypertrophy [43, 121].

In LV and RV failure, alterations in ECC and relaxation are observed. In the LV, diastolic dysfunction with a slower contraction-relaxation kinetic is produced, as well as loss of T-tubules, reduced SR density, and altered Ca^2+^ release from the SR. These changes were also described in RV failure, where loss of T-tubules, smaller and slower intracellular Ca^2+^ transients, reduction and disorganization of the RyR2 network, and reduction of SERCA have been observed in severe hypertrophy caused by MCT-PAH [62]. However, it has been reported that remodeling of the LV wall, which causes diastolic dysfunction, is compensated by an increase in the contraction-relaxation kinetic in cardiomyocytes [122]. PAH-RV treated with resveratrol significantly improves cell relaxation dynamics by enhancing SERCA activity and maintaining the mitochondrial energy supply [121].

The role of Ca^2+^ signaling has been well demonstrated. Ca^2+^ binds to calmodulin (CaM), which activates calcineurin, a phosphatase that dephosphorylates NFAT in the cytosol, allowing its nuclear translocation to regulate the expression of prohypertrophic genes, such as the *β*-myosin heavy chain (*β*-MHC). Additionally, Ca^2+^/CaM activates Ca^2+^/CaM kinase II (CaMKII), which induces the nuclear export of histone deacetylase 5 (HDAC5), derepressing the prohypertrophic transcription factor Mef2 [123]. Mef2 has been implicated in the underlying mechanisms that cause a switch from compensated to decompensated hypertrophy in the RV; Mef2 increases in compensated hypertrophy and decreases during decompensation [124]. In the LV, the role of TGF-*β* and its signaling pathway as a molecular switch has also been reported [65].

## 4. A Clinical and Experimental Therapeutic Approach to RV Dysfunction

As mentioned previously, patients with PAH develop RV remodeling due to the progressive increase in pulmonary vascular resistance and pulmonary artery pressure, leading to RV failure. Although RV failure is the leading cause of death in PAH patients, most PAH treatments (e.g., prostaglandin analogs, Ca^2+^-antagonists, endothelin receptor antagonists, and nitric oxide) target vascular abnormalities. Therefore, the amelioration of RV remodeling and dysfunction may represent an essential aspect of PAH therapy, but unfortunately, current therapies do not improve RV function.

**Figure 2:**
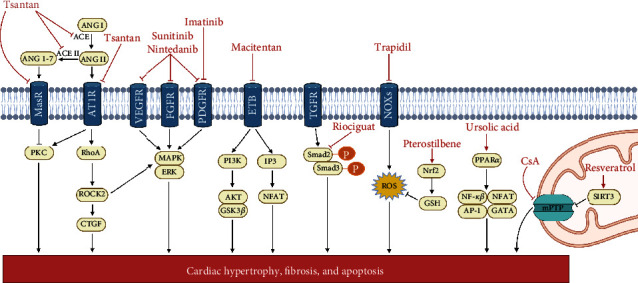
Mechanisms of cardioprotection in *in vivo* therapeutic approaches. Molecules and their effects are shown in red. Lines with arrows indicate activation/stimulation; lines with blunt ends indicate inhibition. Mechanism involved (1) surface receptors blockade: tsantan, sunitinib, nintedanib, imatinib, macitentan, and riociguat; (2) reactive oxygen species inhibition: trapidil and pterostilbene; (3) metabolic capacity preservation: ursolic acid, cyclosporin A (CsA), and resveratrol. ANG: angiotensin; ACE: angiotensin-converting enzyme; MasR: Mas receptor; AT1R: angiotensin II type 1 receptor; VEGFR: vascular endothelial growth factor receptor; FGFR: epidermal growth factor receptor; PDGFR: platelet-derived growth factor receptors; ETB: endothelin receptor type B; TGFR: transforming growth factor receptor; NOXs: nicotinamide adenine dinucleotide phosphate-oxidases; PKC: protein kinase C; ROCK: rho-associated protein kinase; CTGF: connective tissue growth factor; MAPK: mitogen-activated protein kinases; ERK: extracellular signal-regulated kinase; PI3K: phosphatidylinositol 3-kinase; AKT: protein kinase B; GSK3*β*: glycogen synthase kinase 3 beta; IP3: inositol trisphosphate; NFAT: nuclear factor of activated T cells; ROS: reactive oxygen species; Nrf2: nuclear factor E2-related factor 2; GSH: glutathione; PPAR*α*: peroxisome proliferator-activated receptor alpha; NF-*κβ*: nuclear factor kappa-light-chain-enhancer of activated B cells; AP-1: activator protein 1; mPTP: mitochondrial permeability and transition pore; SIRT3: sirtuin 3. The figure was created with BioRender.com.

Under the experimental therapeutic side, different research groups have focused on observing the effects of PAH treatment directly on RV function, mostly using the *in vivo* induction of PAH by MCT, PAB, or hypoxia. Three main action mechanisms are identified. The first and most common mechanism is the blocking of surface receptor signaling, in which the compounds tend to act on multiple receptors. Second, the reduction of cytosolic ROS production by trapidil and pterostilbene at different levels of signaling prevents RV remodeling. The third mechanism is the preservation of the metabolic capacity of the cell, where ursolic acid prevents lipotoxicity, while CsA and RES preserve mitochondrial function.  Table 2 and Figure 2 summarize the effects of different PAH treatments on RV remodeling and protection. Using recombinant human neuregulin (rhNRG-1), Adão et al. observed attenuation in the increased PLB phosphorylation and decreased mRNA expression of *Col1a2*, *Col3a1*, and *ACTA1* caused by MCT-PAH in Wistar rats, as well as decreased passive tension in the rats' isolated RV cardiomyocytes [125]. Treatment with rhNGR-1 also decreased the Fulton index scores, the cardiomyocyte cross-sectional area, and fibrosis caused by PAB-induced PAH in Wistar rats [125]. During treatment of PAH caused by MCT in Sprague-Dawley rats, An et al. [126] observed that a maxingxiongting mixture (MXXTM, an effective Chinese medicine compound prescribed for pulmonary hypertension) reduced the protein expression of RhoA and ROCK II, suggesting that it might improve RV hypertrophy by inhibiting the Rho-kinase signaling pathway in the treatment of pulmonary hypertension. Using a hypoxia-induced PAH *in vivo* model, Dang et al. observed decreased myocardial and RV hypertrophy and collagen deposition, as well as the downregulation of collagen I and III genes and ACE, AngII, and AT1R proteins, when Sprague-Dawley rats were treated with Tsantan Sumtang, a traditional and commonly prescribed Tibetan medicine. Treatment with Tsantan Sumtang attenuated RV remodeling and fibrosis, likely through disruption of the ACE-AngII-AT1R equilibrium in the RV [127].

After treating of PAH caused by an MCT *in vivo* model with ursolic acid, Gao et al. observed the preservation of RV function and the attenuation of hypertrophy indexes. The expression of *Col1a1*, *Col3a1*, *TGFβ1*, and *Bax*, as well as fibrosis and apoptosis markers, also decreased [128]. Similarly, when using resveratrol, a phenolic compound with known cardioprotective effects, in an MCT-PAH *in vivo* model, Vázquez-Garza et al. observed improved RV function measured by tricuspid annular plane systolic excursion (TAPSE) technique and RV free wall thickness and contractility and decreased RV fibrosis and cardiomyocyte area and volume, caused by the low mRNA expression of *BNP*, *Tnnc1*, and *Col1a1*, as well as increased IL-10 and SIRT1 mRNA levels [43]. These mRNA markers were also decreased after using nintedanib to treat PAH in a SU5416+hypoxia *in vivo* model. Rol et al. observed a decrease in RV hypertrophy and collagen content, accompanied by reduced mRNA levels of *Col1a1*, *BNP*, and *OPN* [129]. Furthermore, Leong et al. observed reduced cardiac remodeling biomarker BNP mRNA levels and serum-NT-pro-BNP levels after treating Wistar-Imamichi rats with imatinib and sunitinib in an MCT-PAH *in vivo* model [130].

On the other hand, in a PAB *in vivo* model, Rai et al. observed the preservation of RV function by the attenuation of the increase in RV end-diastolic/systolic volume and collagen content after C57Bl/6J mice were treated with riociguat or sildenafil [131]. These compounds also reduced collagen production and secretion and the phosphorylation of Smad2 and Smad3 proteins when used to treat RV cardiac fibroblast stimulated with *TGFβ1 in vitro*. Therapy with macitentan also improved RV function and hypertrophy caused by PAB in Wistar-Tokyo rats [132].

In a hypoxia-induced PAH model, Schmuck et al. observed that mesenchymal stem cells had a protective effect on RV function. A reduction in RV hypertrophy was observed, with an attenuated RV stroke volume and cardiac output, maintained RV contractility, reduced RV collagen content, and slowed cardiomyocyte enlargement [133].

Poststress conditions could increase ROS in the RV, a determinant factor in many diseases' progression and severity. Pterostilbene complexed with hydroxypropyl-*β*-cyclodextrin (HP*β*CD) to treat PAH induced by MCT *in vivo*, Lacerda et al. observed an increase in GSH concentrations and GSH/GSSG ratio, accompanied by restored glutathione reductase, glutathione-S-transferase, and glutaredoxin enzyme activity [134]. This treatment also increased the expression of SERCA. Similarly, using trapidil to treat PAH in a MCT *in vivo* model, Türck et al. observed increased GSH/total glutathione ratio, decreased NADPH oxidase activity, and increased RV SERCA and RyR protein content [135]. The results of these studies suggest that oxidative stress and improving the RV's Ca^2+^ handling mechanisms may represent valuable targets to treat PAH.

As mitochondria play a key role in heart pathophysiology, Lee et al. investigated the effect of cyclosporine A (CsA) in MCT-PAH *in vivo* [114]. Despite the increase in RV mass, CsA prevented the mitochondrial disruption caused by MCT and attenuated the increases in apoptotic protein Casp3 and Apoptosis-Inducing Factor (AIF) levels. Similarly, Bernal-Ramírez et al. [121] showed that resveratrol treatment in MC-induced PAH rat model avoids mitochondrial permeability and transition pore formation by decreasing cyclophilin D (CypD) hyperacetylation via SIRT3 activation. The combinatorial treatment with macitentan and tadalafil used by Mamazhakypov et al. in Wistar-Kyoto rats with PAH induced by SU5416+hypoxia improved RV function and decreased the expression of hypertrophy A-type natriuretic peptide precursor (NPPA), B-type natriuretic peptide precursor (NPPB), and fibrosis *Col1a1* markers [132]. The results of these studies suggest that the use of combinatorial treatments or the addition of an RV-targeted therapy, such as CsA, might be a new therapeutic strategy in the treatment of PAH.

Various clinical trials have been conducted to better understand or more effectively treat PAH. However, most of them managed cardiac improvement secondary to reduced pulmonary artery pressure instead of managing it as a primary objective. Clinical trials aimed at RV function have studied functional and structural improvement by measuring various parameters.  Table 3 summarizes the available clinical trial results with measures focused on RV function through RVEF, RV end-diastolic, end-systolic volume (RVEDV and RVESV), mass, longitudinal strain, TAPSE, or Tei index parameters.

Among the therapeutic compounds studied for PAH treatment at clinical trials are anti-ischemic agents (e.g., trimetazidine and ranolazine), vasodilators (e.g., treprostinil, sildenafil, tadalafil, and riociguat), endothelin receptor antagonists (e.g., ambrisentan, macitentan, and ambrisentan), beta-blockers (e.g., bisoprolol and carvedilol), stem cells (allogeneic human cardiosphere-derived stem cells), and others. Some of these clinical trials report improvements in RVEF at different percentages: 3.9% after three months of trimetazidine treatment (NCT03273387), 10.4% after six months of carvedilol (NCT00964678) oral treatment, 10.14% after six months of macitentan (NCT02310672) oral treatment, 7.65% and 5.8% after six months of ranolazine (NCT02829034, NCT01839110) oral treatments, and 7.54% after six months of treprostinil inhalations combined with oral tadalafil treatment (NCT01305252). Besides, some clinical trials describe changes in TAPSE such as 7% from baseline after three months of anastrozole treatment (NCT01545336), a decrease from 1.88 to 1.79 cm after four months of QCC374 therapy (NCT02927366), and a decrease from 2.2 to 1.65 cm after nine months of tadalafil and ambrisentan combinational treatment (NCT01042158). These changes reported in RVEF and TAPSE suggest an improvement of the RV function.

Changes in RV volume parameters were also observed in different clinical trials. Treatment with macitentan caused changes in RVSV of 15.17 mL, RVEDV of -6.22 mL, and RVESV of 16.39 mL (NCT02310672); besides, treatment with carvedilol caused a difference of 22.6 mL in RVESV (NCT00964678); these results were observed after six months of treatment with each compound. Along with these changes, improvements in RV mass were reported. Treatment with macitentan for six months caused a reduction of 10.10 g in RV mass (NCT02310672). Similarly, the combinatorial treatment with tadalafil and ambrisentan caused a change in RV mass from 32.5 to 28 g after nine months of treatment (NCT01042158), indicating an improvement in right ventricular remodeling.

As mentioned, most of the conducted clinical trials focus on enhancing cardiac function as a consequence of an improvement in pulmonary artery condition. The clinical trials mentioned here reported improvement in cardiac function with RV function parameters. Unfortunately, these clinical trials aimed at measuring RV function are not primarily focused on molecular parameters, such as mRNA or protein markers of RV damage, which could be helpful in achieving a better understanding of PAH improvement in humans.

### 4.1. Biomarkers in Right Ventricular Dysfunction

Although the clinical trials described above focus on evaluating improvement in the functional parameters of the RV after administering treatment, some studies have evaluated molecular parameters that could be taken into account when evaluating the results of these treatments. Although most of these molecular parameters are not exclusive to right ventricular dysfunction, they could be used as a complementary tool in functional evaluations to determine the diagnosis and prognosis of RV dysfunction. For example, myocardial fibrosis is a hallmark of ventricular remodeling and can be detected by assessing myocardial interstitial collagen content. Although endocardial tissue biopsy is the gold standard in the diagnosis of myocardial fibrosis, researchers have proposed assessing a number of circulating biomarkers with serum analysis markers of collage type I and III turnover, such as procollagen type III amino-terminal propeptide (PIIINP), collagen type I carboxy-terminal telopeptide (CITP), and procollagen type I N-terminal propeptide (PINP), which might serve as surrogate estimates in approximating the intensity of fibrosis in the myocardium. PIIINP may also be a good indicator for right ventricular (SRV) remodeling [136, 137]. Furthermore, cartilage intermediate layer protein 1 (CILP1), an extracellular matrix (ECM) protein involved in profibrotic signaling in the myocardium [138], was recently described as a novel biomarker of RV and LV pathological remodeling in patients with pulmonary hypertension. In one study, maladaptive RV patients had higher CILP1 concentrations than controls and those with LV hypertrophy and dilated cardiac myopathy in [139]. Previously, expression at the RNA level in heart mouse models was shown to be more pronounced in RV pressure overload than in LV pressure overload [140]. Another novel biomarker has been reported; fetal tenascin-C (Tn-C) variants (B+ and C+) are significantly elevated in patients with pulmonary hypertension and can be used to estimate both pulmonary vascular remodeling and RV load in patients with pulmonary hypertension [141]. Furthermore, in an animal model with monocrotaline-induced PAH, Tn-C overexpression has been demonstrated in cardiac tissue [142, 143]. Moreover, it has been reported that elevated serum Interlukin-6 (IL-6) levels in pulmonary hypertension patients are associated with RV dysfunction, regardless of the burden of pulmonary vascular disease. The association between serum IL-6 levels and RV dysfunction may explain the increased mortality in pulmonary hypertension patients with elevated serum IL6 levels, but it is important to clarify that IL6 levels are also related to functional impairment in patients with left ventricular systolic heart failure[144]. BNP and N-terminal pro-BNP are the most commonly used biomarkers in PAH. Both hormones are measurable in plasma and serve as biomarkers of RV dysfunction [145]. However, they are not specific to RV damage and can be elevated in almost all heart diseases [146].

Additionally, cardiac troponin T (cTnT) is used less frequently but has been identified as an independent marker of mortality in patients with PAH [147]; cTnT levels must be correlated with functional and hemodynamic measures [148]. Currently, miRNAs are also important candidate biomarkers. Notwithstanding, most of the upregulated miRNAs in RV failure are similar to those in LV afterload stress. In a murine model of right ventricular hypertrophy (RVH) and right ventricular failure (RVF) using pulmonary artery constriction, RV-specific miRNAs 34a, 28, 93, and 148a were found; however, none of these are increased in LV hypertrophy and failure induced by transverse aortic constriction [149]. Interestingly, a transcriptomic study of human RVH via RNA expression and network analysis using exclusively freshly isolated myocardium of pediatric patients with tetralogy of Fallot/pulmonary stenosis found that miR-371a and miR-372 are differentially expressed when compared to controls. The authors suggest that these miRNAs are potential biomarkers for diseases associated with RV pressure overload [150]. In a study with 40 patients with RV pressure overload by pulmonary hypertension, it was reported that circulating levels of miR-21, miR-130a, miR-133b, miR-191, miR-204, and miR-208b were higher, while the levels of miR-1, miR26a, miR-29c, miR-34b, miR-451, and miR-1246 were lower in comparison to matched controls. This study also confirmed that a correlation exists between the severity of PAH and circulating levels of miR-133b and miR-208b with [151]. It was also shown that long noncoding RNA H19 is upregulated in decompensated RV from PAH patients and correlates with RV hypertrophy and fibrosis. These findings were corroborated in a rat model of monocrotaline and pulmonary artery banding. The authors propose that H19 is a promising biomarker for the prognosis and severity of RV dysfunction by PAH [152].

Additional research is needed to identify new biomarkers that can further improve diagnostic accuracy. It is important to define standard operating procedures for blood and tissue collection, processing, and storage, as well as miRNA analysis, to ensure precise quantification. Additionally, it is necessary to correlate all emerging biomarkers with right ventricular function for robust validation.

## 5. Final Thoughts

The ventricles have commonly been studied as individual entities; however, each ventricle must adapt its function to perform its respective role in coordination with each other. Structural differences between ventricles determine their physiological differences in function at the organ level. LV contraction involves radially constricting and longitudinally shortening the septum, while RV contraction is more passive since the RV's free wall lies flat against the septum when LV contracts. The prolonged AP in the LV and the slower contraction velocity in the RV may be a mechanism to coordinate whole-organ contraction. Changes in ECC may compensate for the differences in muscle thickness and ejection pressure to allow ventricles to synchronize at the end of the systole. Furthermore, the discrepancies reported in changes at different stages of the ECC may be due to the heterogeneity within and between ventricles [26, 31, 153]. Physiological differences in the structure, function, and molecular adaptations of the LV and RV result in different responses to stressful stimuli. While LV thickness helps to support higher pressures, a thin RV wall is highly susceptible to increases in vascular resistance. A better understanding of the differences in cellular and molecular alterations in the LV and RV due to remodeling and failure may facilitate the development of more effective therapeutic approaches. This understanding is especially important for the RV, given that the mechanisms related to its dysfunction are not as widely studied as those related to LV's dysfunction.

Advances in RV dysfunction diagnostics and therapeutics are needed to improve the early detection of the disease and improve prognosis. The determination and validation of new, less-invasive, and more accurate biomarkers is an important research area that has been strengthened by the description of ventricle differences in dysfunction. Additionally, the search for new therapeutic approaches that target RV has been fueled by the variation in response between the two ventricles. Some diverse therapeutic strategies could be beneficial in improving current treatments (Table 4), but they require further investigation to estimate their contribution to patient morbidity and mortality.

## Figures and Tables

**Table 1:** Physiological differences between ventricles in myocyte function.

Process	Component	Level	RV change (Compared to LV)	Model	Reference
Action potential	INa	Density	Lower	Dog	[26]
		Expression (SCN5A,SCN1B and 4B)	NC	Dog	
		Steady-state inactivation	Higher	Dog	
		Recovery from inactivation	Higher	Dog	
	AP	Duration	Higher	Human, Dog, Rat, Mice, Human	[29, 30, 32, 34–37]
			NC	Guinea pig	[38]
	Ito	Current	Higher	Dog, Mice, Rat, Dog	[28, 30–32]
		Expression	NC	Rabbit, Mice	[31, 33]
		Inactivation constant	NC	Dog, Dog	[30, 32]
	ICa	Current	NC	Mice	[29]
	IKs	Density	Higher	Dog	[32]
		Expression	NC	Rabbit	[33]
	IKr	Density	NC	Dog	[32]
	IK1	Expression	NC	Mice, Dog	[30, 31]
		Density	NC	Mice	[29]
	ISS	Density	NC	Dog, Mice	[30, 31]
	IKATP	Expression	Lower	Guinea pig	[38]

CIRC	LTCC	Expression	Higher	Rabbit	[27]
			NC	Mice	[29]
		Current	NC	Mice	[29]
	RyR	Activity	NC	Human	[39]
		Sensitivity	NC	Human	[39]
		Density	NC	Human	[39]
		Expression	NC	Rabbit, Human	[27, 154]
			Lower	Dog	[40]
	Ca^2+^ transient	Amplitude	Lower	Rat, Mice	[29, 42]
		Time to decay	Higher	Rat	[36]
	SR	Volume	NC	Pig	[54]
		Ca^2+^ load	Lower	Rat	[42]
	Diastolic Ca^2+^	Level	NC	Mice, Rat	[29, 41]

Cell contraction	Contraction force		Lower	Dog	[36]
	Sarcomere shortening		Lower	Rat, Mice, Dog, Rat	[29, 36, 41, 43]
	Troponin I	Phosphorylation	NC	Mice	[46]
	Troponin T	Phosphorylation	NC	Mice	[46]
	MyBP-C	Phosphorylation	NC	Mice	[46]
	MRLC	Phosphorylation	NC	Mice	[46]
	Actin-Myosin binding	Mobility	Lower	Mice, Rabbit	[44, 45]
	Maximal shortening velocity		Lower	Mice	[29]
	Myofilaments Ca^2+^ sensitivity		Lower	Rat, Mice	[46–48]
	Myosine ATPase activity		Higher	Rat, Rat	[49, 50]
	Myosine heavy chain	Alfa: beta proportion	Higher	Rat	[49]

Cell relaxation	SERCA	Activity	Lower	Rat, Rat	[41, 42]
			Higher	Rat	[36]
			NC	Mice	[29]
		Expression	Lower	Rat	[41]
			NC	Rat, Rabbit	[27, 42]
		Phosphorylation	Lower	Rat	[41]
		Affinity to Ca^2+^	Lower	Rat	[41]
	SERCA-PBL	Ratio	NC	Rat	[41]
		Stability complex	Higher	Rat	
	NCX	Expression	Higher	Rabbit	[27]
		Rest-potentiation phenomenon	Higher	Rat, Mice	[29, 36]

Cell energetics	Mitochondria respiration	Expression	NC	Rat	[53]
		Activity	NC	Dog	[52]
	Oxidative metabolism	Expression	NC	Rat	[53]
	Fatty acid oxidation	Expression	NC	Rat	[53]
	Rate of oxidation	Activity	Lower	Rat	[53]
	Mitochondria content	Citrate synthase activity	Lower	Rat	[53]
		Mitochondria-myofibrils ratio	Lower	Pig	[54]
		Mitochondria volume	NC	Pig	[54]

NC: no change.

**Table 2:** Treatment of PAH focused on RV remodeling and protection of its function.

Biological subject	Treatment	Experimental model	Effect on RV compared with the model group	Reference
Isolated skinned cardiomyocytes (Wistar rats)	Recombinant human neuregulin-1 (rhNRG-1)	MC-induced PAH	Decreased RV isolated cardiomyocyte passive tension	[125]
Wistar rats	Recombinant human neuregulin-1 (rhNRG-1)	MC-induced PAH	Attenuate the increase of phospholamban phosphorylation Attenuate the upregulated mRNA expression of *Col1a2*, *Col3a1*, and *ACTA1*	[125]
		PAB-induced pressure overload	Decreased Fulton index, cardiomyocyte CSA, and fibrosis	
Sprague-Dawley rats	Maxingxiongting mixture	MC-induced PAH	Attenuate the upregulated protein expression of RhoA and ROCK II	[126]
Sprague-Dawley rats	Tsantan Sumtang	Hx-induced PAH	Decrease RVHI, RV/TL, myocardial hypertrophy, and collagen deposition Downregulate collagen I and III levels and hydroxyproline content Downregulated levels of ACE, AngII, and AT1R proteins	[127]
Sprague-Dawley rats	Ursolic acid	MC-induced PAH	Higher TAPSE and PAT/PET Prevented increase in RVSP Attenuated the increase of RVHI, RV myocardial cell size, and cross-sectional area Attenuated the increased expression of *Col1a1*, *Col3a1*, *TGFβ1*, and *Bax*	[128]
Sprague-Dawley rats	Resveratrol	MC-induced PAH	Improved TAPSE, RV free wall thickness, and contractility Decreased RV fibrosis and cardiomyocyte area and volume Decreased BNP, Tnnc1, and Col1a1 mRNA levels Increased IL-10 and SIRT1 mRNA levels	[43]
Sprague-Dawley rats	Nintedanib	SU5416+Hx-induced PAH	Decreased RV hypertrophy Reduced RV total collagen content Reduced *Col1a1*, *BNP*, and *OPN* mRNA levels	[129]
Wistar-Imamichi rats	Imatinib	MC-induced PAH	Reduced RVH Reduced RV BNP mRNA expression and serum NT-pro-BNP levels	[130]
	Sunitinib		Reduced RVH Reduced RV BNP mRNA expression and serum NT-pro-BNP levels	
C57Bl/6J mice	Riociguat	PAB-induced pressure overload	Attenuated the increase of RV end-diastolic/systolic volume Reduced RV collagen content	[131]
	Sildenafil		Attenuated the increase of RV end-diastolic/systolic volume	
Sprague-Dawley rats	Mesenchymal stem cells	SU5416+Hx-induced PAH	Reduced RV hypertrophy Attenuated the reduction of RV stroke volume and cardiac output Maintained RV contractility Reduced RV collagen content and cardiomyocyte enlargement	[133]
Wistar-Kyoto rats	Macitentan	SU5416+Hx-induced PAH	Reduced RVSP, TPVR, and RV hypertrophy Increased cardiac output, TAPSE, and RV dilatation Attenuated the increase of NPPA and NPPB expression Attenuated the increase of *Col1a1*	[132]
	Tadalafil		Reduced RVSP, TPVR, and RV hypertrophy Increased cardiac output, TAPSE, and RV dilatation Attenuated the increase of NPPA and NPPB expression Attenuated the increase of *Col1a1*	
	Macitentan+tadalafil		Reduced RVSP, TPVR, and RV hypertrophy Increased cardiac output, TAPSE, and RV dilatation Attenuated the increase of NPPA and NPPB expression Attenuated the increase of *Col1a1* and *Col3a1*	
Wistar rats	Pterostilbene complexed with HP*β*CD	MC-induced PAH	Increased concentration of GSH and GSH/GSSG ratio Restored the activity of GSR, GST, and GRx Reduced TBARS levels Increased expression of SERCA	[134]
Wistar rats	Trapidil	MC-induced PAH	Increased GSH/total glutathione ratio Decreased NADPH oxidase activity Increased RV SERCA and ryanodine receptor protein content	[135]
Sprague-Dawley rats	Cyclosporine A	MC-induced PAH	Increased RV mass Prevented mitochondrial disruptions Attenuated the increase of Casp3 and AIF protein levels	[114]
Sprague-Dawley rats	17*β*-Estradiol	MC-induced PAH	Reduced RV diameter, wall thickness, fibrosis, RV/LV+IVS, and RV/BW ratio Improvement of TAPSE, RVFAC, and RIMP Decreased serum BNP levels	[155]

**Table 3:** Clinical trials with aim in measuring RV function.

Clinicaltrials.gov identifier	Trial status	Intervention	RV outcome measures	Results
NCT03273387	Completed	Trimetazidine	Changes in RV ejection fraction after 3 months	Improvement of RVEF (3.9%) from baseline
NCT03835676	Recruiting	Treprostinil	Effects on right ventricular structure and function using echocardiography Effects on right ventricular structure and function using cardiac magnetic resonance imaging	No results reported
NCT02253394	Terminated (low enrollment)	Ambrisentan plus spironolactone	Effect on cardiac output	No results reported
NCT04435782	Not yet recruiting	JNJ-67896049	Change from baseline to week 26 in RVSV, RVEDV, RVESV, RVEF, mass, and RVGLS in participants will be assessed by pulmonary artery flow MRI	No results reported
NCT02074449	Completed	Treprostinil	Change in RV coupling index between baseline, titration at 48-72 hours, and 3 months	No results reported
NCT01545336	Completed	Anastrozole	Tricuspid annular plane systolic excursion (TAPSE) from baseline to 3 months	7% change from baseline
NCT02310672	Completed	Macitentan	Change from baseline in RVSV, RVEDV, RVESV, RVEF, and mass to week 26	Change of 15.17 mL of RVSV, -6.22 mL of RVEDV, -16.39 mL of RVESV, 10.14% of RVEF, and -10.10 g to week 26
NCT02169752	Terminated (PI left National Jewish Health)	Ambrisentan	Improvement in RV myocardial strain from baseline to 1, 3, and 6 months	No results reported
NCT03236818	Unknown	ERA and PDE-5I (sildenafil, tadalafil, bosentan, macitentan)	Change in RVEF	No results reported
NCT01083524	Completed	Dichloroacetate sodium	Changes in RV size/function	No results reported
NCT01246037	Unknown	Bisoprolol	Improvement of maladaptive remodeling of the RV wall and diastolic properties of RV	No results reported
NCT00742014	Suspended (absorption of oral sildenafil not consistent)	Sildenafil	Increase in end-systolic elastance of the right ventricle from baseline	No results reported
NCT01148836	Completed	Coenzyme Q-10 Dietary supplement	RV outflow and myocardial performance from baseline to 3 months	RV outflow from 11.3 to 13.5 cm and performance ratio from 0.9 to 0.7
NCT01757808	Completed	Ranolazine	Change in RV echo parameters	No results reported
NCT03617458	Recruiting	Metformin	Change from baseline to week 12 in RV myocardial muscle triglyceride content, TAPSE, RVEF, RV fractional area, RV diastolic function, and RV free wall longitudinal strain	No results reported
NCT04062565	Recruiting	Treprostinil	Change in RV diastolic stiffness	No results reported
NCT02829034	Completed	Ranolazine	Change from baseline in RVEF to 26 weeks	Change of 7.56% from baseline
NCT01839110	Completed	Ranolazine	Changes from baseline in RVEF to 6 months	Change of 5.8% from baseline
NCT02939599	Terminated (study was terminated early for strategic reasons; only part I of the study was completed)	QCC374	Change from baseline in RV Tei index and RV fractional area at week 16	Tei index change of 0.84 and fractional area of 23.91%
NCT02927366	Terminated (study was terminated early for strategic reasons; only part I of the study was completed)	QCC374	Change from baseline in RV fractional area, Tei index, and TAPSE	Change from 20.17 to 20.70% of fractional area, 0.92 to 0.89 of Tei index, and TAPSE from 1.88 to 1.79 cm
NCT00964678	Completed	Carvedilol	Change from baseline in RVEF and RVESV to 6 months	Change in RVEF of 10.4% and RVESV of 22.6 mL
NCT03344159	Suspended (COVID-19 pandemic)	Spironolactone	Change from baseline of RV wall stress, structure, function, and area of fibrosis	No results reported
NCT02507011	Terminated	Carvedilol	Mean change in RVEF	Change in RVEF of 10%
NCT01174173	Completed	Ranolazine	Change from baseline in absolute RV longitudinal strain to 3 months	Change in RV longitudinal strain from -1.4 to 1.0%
NCT02744339	Completed	Riociguat	Change from baseline in RVEF and RV volume to 26 weeks	No results reported
NCT02102672	Unknown	Trimetazidine	Change from baseline in RV function to 3 months	No results reported
NCT03648385	Recruiting	Dehydroepiandrosterone	Chance from baseline in RV longitudinal strain and RVEF to 40 weeks	No results reported
NCT01042158	Completed	Tadalafil and ambrisentan	Change from baseline in RV mass and TAPSE to 36 weeks	Change in RV mass from 32.5 to 28 g and TAPSE from 2.2 to 1.65 cm
NCT03145298	Recruiting	Allogeneic human cardiosphere-derived stem cells	Change in RV ventricular function	No results reported
NCT03362047	Recruiting	Riociguat and macitentan	Change from baseline in RV function and contractility to 12 weeks	No results reported
NCT03449524	Terminated	CXA-10	Change from baseline in RVEF to 6 months	No results reported
NCT01305252	Completed	Treprostinil inhalations and tadalafil	Change from baseline in RVEF to 24 weeks	Change of 7.45% in RVEF
NCT01917136	Completed	11C-acetate and [18F]fluoro-2-deoxy-2-D-glucose	Change from baseline in RVEF to 6 months	Change of 7.56% of RVEF
NCT00772135	Unknown	Sildenafil citrate	Change from baseline in RV pressure	No results reported

**Table 4:** Possible therapeutic strategies to address key alterations in RV versus LV dysfunction.

	Possible therapeutic strategies	Ref.
Fibrosis	Current antifibrotic therapies effective in LV do not reverse RV fibrosis, possibly due to differences in ECM composition.	[69, 70, 156–159]
Myocyte contraction	There is improvement of sarcomere function by PKA activators since RV myofilaments have lower Ca^2+^ sensitivity.	[46–48, 160, 161]
Inflammation	RV has more macrophages and dendritic cells, which could mean inflammation plays a more important role.	[162, 163]
Mitochondrial dynamics	PAH presents excessive RV mitochondrial fission, which could indicate significant mitochondrial quality control impairment.	[164–166]
Mitochondrial function	RV has less mitochondrial content and a lower rate of oxidation; thus, the preservation of mitochondrial integrity and membrane potential improves RV function.	[43, 53, 121, 167]

## Data Availability

The data used to support the findings of this study are available from the corresponding authors upon request.
